# COMMUNICATION WITH HEALTHCARE PROVIDERS DURING THE PANDEMIC: EXPERIENCES OF OLDER ADULTS

**DOI:** 10.1093/geroni/igac059.2223

**Published:** 2022-12-20

**Authors:** Logan Sweeder, Andrea Sillner, Marie Boltz, Kimberly Van Haitsma

**Affiliations:** The Pennsylvania State University, State College, Pennsylvania, United States; Pennsylvania State University Ross and Carol Nese College of Nursing, University Park, Pennsylvania, United States; Penn State, Pennsylvania State University, Pennsylvania, United States; Pennsylvania State University, University Park, Pennsylvania, United States

## Abstract

The COVID-19 pandemic introduced many societal changes, including the need to use technology to communicate with healthcare providers. For many this was a new form of communication. This study examines (1) who older adults identified as healthcare providers and (2) reflected on how communications with formal healthcare providers changed during the pandemic. This data is a subset of a cross-sectional descriptive survey that was distributed electronically to a sample of community-dwelling older adults aged 65 years and older (N=323). Qualitative data was analyzed using thematic analysis. Quantitative data was analyzed using descriptive statistics. Participants averaged 72.7 years of age and 74.5% were retired. Just over ¼ of the sample identified nurses as part of their formal healthcare team; however, participants who identified nurses as formal healthcare providers had fewer negative themes regarding telehealth communication with healthcare providers compared to those who did not. They were also more likely to support in-person and technology driven communication models in the future. Telehealth remains an evolving form of primary healthcare that was utilized throughout the COVID-19 pandemic. Many older adults in our study considered it a resource that requires further optimization while others were staunch opponents to its continued employment in healthcare settings. Nurses are a vital part of our healthcare system and frequently interact with patients and families to coordinate care. Further research should examine how different scopes of nursing practice can support the implementation of telehealth to improve care delivery among community-dwelling older adults.

